# Efficiency of mitigation measures targeting nutrient losses from agricultural drainage systems: A review

**DOI:** 10.1007/s13280-020-01345-5

**Published:** 2020-06-03

**Authors:** Mette Vodder Carstensen, Fatemeh Hashemi, Carl Christian Hoffmann, Dominik Zak, Joachim Audet, Brian Kronvang

**Affiliations:** Department of Bioscience, Aarhus University, Vejlsøvej 25, 8600 Silkeborg, Denmark

**Keywords:** Agricultural drainage systems, Catchment management, Meta-analysis, Mitigation measures, Nutrient reduction, Water quality

## Abstract

Diffusive losses of nitrogen and phosphorus from agricultural areas have detrimental effects on freshwater and marine ecosystems. Mitigation measures treating drainage water before it enters streams hold a high potential for reducing nitrogen and phosphorus losses from agricultural areas. To achieve a better understanding of the opportunities and challenges characterising current and new drainage mitigation measures in oceanic and continental climates, we reviewed the nitrate and total phosphorus removal efficiency of: (i) free water surface constructed wetlands, (ii) denitrifying bioreactors, (iii) controlled drainage, (iv) saturated buffer zones and (v) integrated buffer zones. Our data analysis showed that the load of nitrate was substantially reduced by all five drainage mitigation measures, while they mainly acted as sinks of total phosphorus, but occasionally, also as sources. The various factors influencing performance, such as design, runoff characteristics and hydrology, differed in the studies, resulting in large variation in the reported removal efficiencies.

**Electronic supplementary material:**

The online version of this article (10.1007/s13280-020-01345-5) contains supplementary material, which is available to authorized users.

## Introduction

The high intensive agricultural production dominating parts of the world, such as Western Europe and North America, is one of the main causes of eutrophication resulting in water quality problems and ecosystem degradation worldwide (Kronvang et al. 2005; Diaz and Rosenberg 2008; Steffen et al. 2015). The intensification and expansion of agriculture during the past decades have led to a drastic increase in nutrient loss from agricultural areas, as well as changes in land use. Wet landscapes have been systematically drained to enable anthropogenic activities such as food production (Skaggs and van Schilfgaarde 1999). However, in addition to water, drainage systems also transport nutrients rapidly to surface waters, thereby lowering the natural retention capacity of catchments. Thus, engineered ecotechnologies designed to intercept and reduce nitrogen (N) and phosphorus (P) losses from agricultural drainage systems have emerged over the last decades with the aim to improve water quality (Mitsch and Jørgensen 1989). Substantial changes in land use can also be expected in the future when addressing energy and food security such as transformation of the society to a bio-economy (Marttila et al. 2020; Rakovic et al. 2020). Water quality and quantity are key elements in such a transformation, thus the development and implementation of drainage mitigation provide valuable opportunities for innovation in future bio-economies. Besides reducing nutrient losses to surface water, these measures can be designed to provide multiple ecosystem services, such as water storage and biomass production, as well as recycling of nutrients.

Drainage mitigation measures reduce the transport of N from drainage systems primarily by enhancing denitrification (O’Geen et al. 2010), i.e. the process by which nitrate dissolved in water is converted to atmospheric nitrogen (Knowles 1982). Denitrification requires anoxic conditions, electron donors and availability of organic carbon. If these requirements are met, the rate of the denitrification is mainly controlled by temperature and the hydraulic retention time (HRT), which is inversely proportional to the water flow rate (Kadlec and Knight 1996; Hoffmann et al. 2019). The water flow from subsurface drainage systems is driven by precipitation and snowmelt and, thus, varies greatly on a temporal as well as a spatial scale (Skaggs and van Schilfgaarde 1999). This challenges the performance of drainage mitigation measures in some parts of the world, for instance the Nordic countries, where high loading rates of nitrate often occur during autumn to early spring when the water temperature and denitrification rates are low. Therefore, we were particularly interested in investigating the nitrate removal efficiency of drainage mitigation measures treating drainage water in climate zones, where high loading rates of nitrate often occur when conditions for denitrification is suboptimal. In addition to nitrate removal, drainage mitigation measures have shown potential for retention of P as increased HRT allows settling of suspended material such as sediment and particulate P (PP). Yet, the anoxic conditions established by these mitigation measures might lead to net P release, depending on local hydrological and geochemical conditions (O’Geen et al. 2010).

**Fig. 1:**
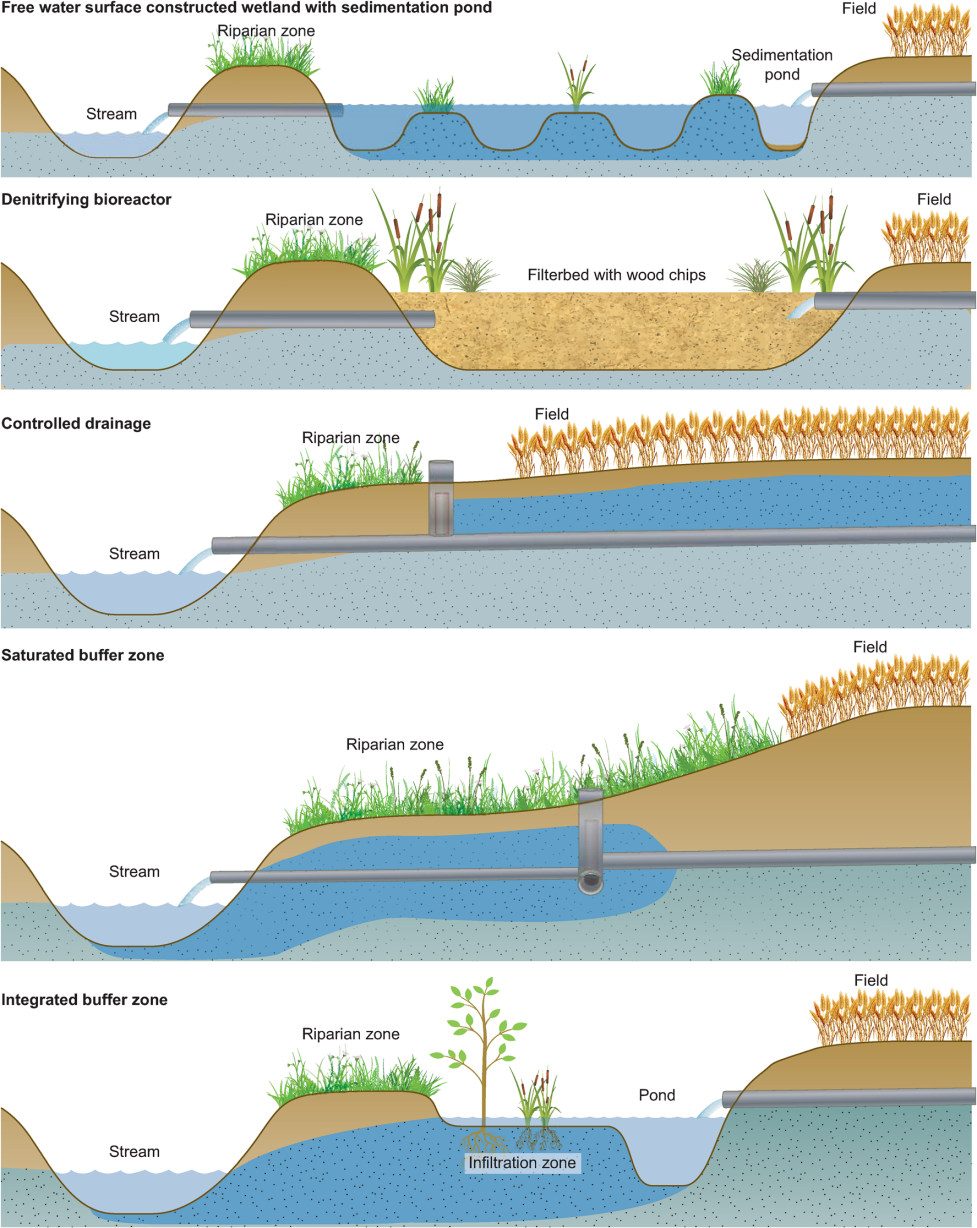
Conceptual scheme of the five drainage mitigation measures

In this review, we focused on five types of mitigation measures treating drainage water before it enters streams. These were the commonly applied free water surface flow constructed wetlands (FWS), denitrifying bioreactors (DBR) and controlled drainage (CD) and the two emergent technologies saturated buffer zones (SBZ) and integrated buffer zones (IBZ) (Fig. 1). To obtain a better understanding of the opportunities and challenges of current and new drainage mitigation measures targeting the transport of nutrients from agricultural areas in oceanic and continental climates, we examined nitrate and total P (TP) removal efficiencies at 82 drainage sites established between 1991 and 2018 in eleven countries. Thus, this review compiles the available evidence on nitrate and TP removal efficiencies from both pilot and full-scale field studies on drainage mitigation measures to provide a synthesis of the existing body of peer-reviewed literature.

## Materials and methods

### Overview of the included types of drainage mitigation measures

#### Free water surface constructed wetlands (FWS)

In FWS, drainage water typically passes one or more deep basins or channels and shallow vegetated zones (berms) before reaching the outlet and eventually the stream (Kovacic et al. 2000) (Fig. 1). The deep zones reduce the water flow and thus increase HRT and promote denitrification and sedimentation, while the shallow vegetation berms supply organic carbon. Furthermore, FWS can capture surface runoff if located downhill. Free water surface constructed wetlands are mostly established in areas with low permeable soils, and if not, they are often sealed with non-permeable layers such as clay membranes to prevent seepage to the groundwater. Construction of wetlands for diffusive pollution control began in the late 1980s with the aim to create simple systems mimicking the processes occurring in natural wetlands (Mitsch and Jørgensen 1989; Fleischer et al. 1994). Multiple types of FWS exist (Mitsch et al. 2001), although in this review, we focused only on the subset of FWS designed to treat drainage water before it reaches streams.

#### Denitrifying bioreactors (DBR)

In DBR, the drainage water is routed horizontally or vertically through a basin filled with carbon-rich filter substrate (e.g. different types of wood chips mixed with gravel, soil or other materials) before it reaches the outlet (Blowes et al. 1994) (Fig. 1). The substrate of the DBR can either be in direct contact with air (David et al. 2016; Carstensen et al. 2019b) or sealed off by a layer of soil on top of the reactor (de Haan et al. 2010). Similar to FWS, the base of the DBR are sealed with non-permeable membranes to avoid seepage if establish on water-permeable soils. Denitrifying bioreactors are also known as subsurface flow constructed wetlands, denitrifying beds or bio-filters. The first pilot study with DBR, established in Canada in 1994, was inspired by wastewater treatment plants (Blowes et al. 1994). However, in contrast to wastewater treatment plants, DBR was solely designed to promote anoxic conditions, and carbon-rich filter material was added to fuse denitrification.

#### Controlled drainage (CD)

Controlled drainage is a groundwater management technique, where the in-field groundwater level is elevated using a water control structure to restrict the water flow from the drain outlet (Gilliam et al. 1979) (Fig. 1). Thus, CD alters the hydrological cycle of the field, which, depending on location and season, increases some or all of the following flow components: root zone water storage, seepage (shallow, deep), surface runoff, plant uptake and evaporation (Skaggs et al. 2012). Experiments with CD were initiated in the late 1970s in the USA to investigate the potential for enhancing in-field denitrification (Willardson et al. 1970), and CD were also practiced in the former German Democratic Republic to cope with summer droughts, though the technique disappeared with the fall of the wall (Heinrich 2012).

#### Saturated buffer zones (SBZ)

In a SBZ, drainage water and riparian soil are reconnected by a buried, lateral perforated distribution pipe running parallel to the stream, which redirect the drainage water into the riparian zone (Jaynes and Isenhart 2019) (Fig. 1). The infiltrating water saturates the riparian soil and creates anoxic conditions, though in order for denitrification to occur, the soil carbon content must be sufficient. This novel technique was recently developed and tested in the USA (Jaynes and Isenhart 2014).

#### Integrated buffer zones (IBZ)

In IBZ, the drainage water is first retained in a pond designed to capture particles and increase the HRT and to buffer surface runoff (Zak et al. 2018) (Fig. 1). After the pond, the water infiltrates a vegetated shallow zone where the top soil has been removed. In this infiltration zone, anoxic conditions develop and carbon is added from the vegetation via root exudates or leached plant litter. Integrated buffer zones were recently developed and tested in Northwestern Europe with the aim to improve the nutrient reduction capacity of traditional riparian buffer zones bypassed by drainage pipes, while promoting multi-functionality, such as biodiversity and biomass production (Zak et al. 2019).

### Literature search and inclusion criteria

**Fig. 2:**
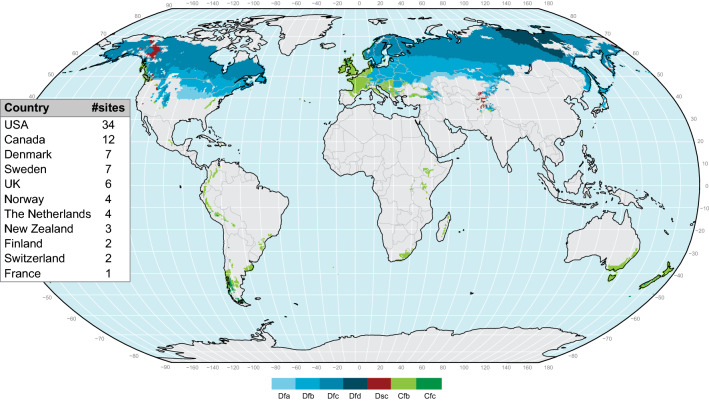
World map showing the climate regions included in the review and the number of study sites per country
- The study had to be a field study with sites exposed to ambient temperature and with a surface area larger than 10 m^2^.
- The study had to include a mass balance for either nitrate—N, total phosphorus (TP) or total suspended solids (TSS) for at least one drainage season, whose length depended on the climate region.

To find relevant studies for our review, a search of published studies was conducted via ISI Web of Science for 1900–2019 employing four different search strings, which are described in the Supplementary Material (Table S1). The relevant studies was selected considering the following criteria:

- The inlet water had to originate from drainage systems transporting water from agricultural fields, and must not be mixed with water from other sources such as streams.
- Based on the Köppen-Geiger climate classification system, the sites had to be located in oceanic (Cfb, Cfc) or continental (Dfa, Dfb, Dfc, Dfd, Dsc) climates (Fig. 2), where the conditions for denitrification are often suboptimal. Thus, climate zones with dry winters (letter w) were excluded.

If two studies were conducted at the same study site within overlapping monitoring periods, the study with the longest time series was selected. Not all extracted data could be separated into years or seasons, implying that standard deviation (σ) for nitrate removal was not available for nine sites and for TP removal for one site; still, these sites were included in the calculation of the arithmetic mean (Table S2). Absolute removal was reported in various units (e.g. g m^−2^, kg ha^−1^, g m^−3^), and we therefore identified and used the most commonly reported unit, which meant that recalculation of removal efficiencies were necessary in some studies.

### Meta-analysis

The average nitrate and TP removal efficiencies of mitigation measures treating agricultural drainage water were quantified using meta-analysis. Prior to the analysis, the assumption of normality was tested visually (Q–Q plot, histogram) and by the Shapiro–Wilk test, and where the assumptions were not fulfilled this is mentioned in the result section. Meta-analysis was only conducted for a mitigation measure if sufficient data were available, i.e. data from more than two sites originating from different studies. The meta-analysis was performed in R software 3.6.1 (R Core Team 2019) using the R package ‘meta’ (Schwarzer 2019). The effect size of each study was expressed as the raw removal efficiency and was calculated as follows:

$${\text{Removal}} \;{\text{efficiency}} \left( \% \right) = \left( {\frac{{{\text{Load}}_{\text{in}} - {\text{Load}}_{\text{out}} }}{{{\text{Load}}_{\text{in}} }}} \right) \times 100$$

where Load_in_ is the loading to the system in kg year^−1^ and Load_out_ the loss from the system in kg year^−1^; for CD sites the unit is kg ha^−1^ year^−1^.

Each effect size was weighted, and a higher weight was given to studies with small standard error (SE) and large sample size, as these were regarded as more precise. The summary effect was calculated based on the effect sizes and their weight, using a random effect model, which allow the true mean to vary between studies, as the selected studies differed in design, materials and methods. To account for this variability, the weighting factor assigned to each effect size incorporated both the within-study variance (σ^2^) and the between-study variance (*T*^2^). The DerSimonian and Laird (DL) method was applied to estimate *T*^2^, and the Hartung-Knapp method was used to adjust the confidence intervals (CI), producing more conservative results, as recommended by Borenstein (2009), when dealing with a low number of studies (*K* < 20). To evaluate whether the use of the overall summary effect was appropriate, the degree of consistency of the effect sizes was assessed using forest plot, funnel plot and multiple statistical measures. The observed variation (*Q*) was tested to investigate if the true effect varied between studies and if application of the random effect model was appropriate (Borenstein 2009). The excess variation over the observed variation (*I*^2^) gave an indication of what proportion of the variation was real, and reflected the extent of overlapping CIs. However, care must be taken, as in the case of an *I*^2^ close to zero, it can either be ascribed to that all variance is due to sampling error within the studies, though it can also be  caused by very imprecise studies with substantial difference between effect sizes. Thus, large *I*^2^ values can either indicate the possible existence of different subgroups or that the analysis contain highly precise studies with very small differences between the effect sizes. The estimate of the absolute variance, *T*^2^, was used as an indication of dispersion, and it was compared with σ^2^. The standard deviation of the effect size (*T*) was also reported. In the funnel plot, the removal efficiencies were plotted against the SE, thus asymmetry or other shapes in the funnel plot might indicate bias related to publication bias, heterogeneity or sampling error. Funnel plots were only inspected if the analysis contained more than ten studies, as recommend by Borenstein (2009). For each effect size and summary effect, a 95% CI was reported. Additionally, a 95% prediction interval (PI) was calculated for each summary effect, yielding the interval where 95% of future studies will fall (Borenstein 2009). To further explore heterogeneity and the robustness of the summary effect, a meta-analysis was performed on two subsets of data for each drainage mitigation measure if data sufficed. The first subset of data contained only sites from the low risk of bias category (“Risk of bias assessment”), while the other data set only contained sites where the within-study sample size (N) was larger than two.

### Risk of bias assessment

**Fig. 3:**
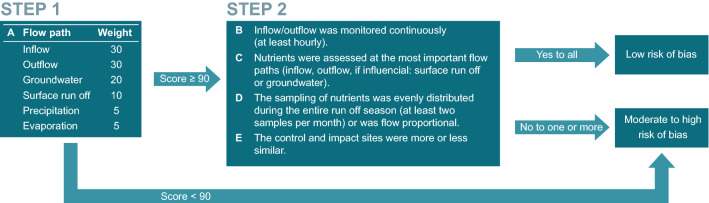
Overview of the method for risk of bias assessment

It is important to consider the extent of systematic errors resulting from different factors such as a poor study design or issues related to the collection, analysis and reporting of data when conducting a review. In this study, the risk of bias tool developed by Higgins et al. (2011) was used as a guideline, although it was originally developed based on evidence from randomised trials within the field of meta-epidemiology. However, it has earlier been modified and used for environmental studies (Bilotta et al. 2014), such as wetlands (Land et al. 2016). In our study, the risk of bias assessment included two steps (Fig. 3), where the first step was an evaluation of the water balance monitoring strategy (1.A in Fig. 3). The water balance is especially of importance when quantifying the removal efficiency, as any errors here will propagate into the nutrient balance. To assess the monitoring strategy of the water balance, the most important flow paths were given a percentage, and aggregated into an overall score. Thus, monitoring of inflow and outflow accounted for 30%, groundwater for 20%, surface runoff for 10% and precipitation and evaporation for 5% each. A percentage of 100% implied that all important flow paths were monitored or otherwise accounted for. In the second step, the monitoring frequency of flow (2.B in Fig. 3) and the spatial and temporal frequency of nutrient sampling (2.C, 2.D) were assessed. Finally, the selection of control and impact sites was evaluated (2.E in Fig. 3); however, this was only relevant for studies on CD, as these were the only studies with true spatial replication. In the remaining studies, the inlet served as control and the outlet as impact. If all five attributes were fulfilled, the site was considered as having low risk of bias; otherwise, it was considered having moderate to high risk of bias.

## Results

### Descriptive characteristics

The initial search yielded 8126 studies in total, and after evaluating the inclusion criteria, we had a master bibliography of 42 articles containing 84 sites distributed across eleven countries (Table 1 and Table S2). According to our risk of bias assessment, the risk of bias was low in 35% of the studies. Insufficient monitoring of the water balance was the main reason that many studies were categorised as having ‘moderate to high’ risk of bias (Table 1). The ratio of the drainage mitigation measure surface area to the contributing catchment area (DMMCAR) was largest for SBZ (7%) and FWS (2%), while DBR (0.1%) and IBZ (0.2%) had the lowest ratios (Table 2). For CD, the DMMCAR was technically 100% if assuming that the groundwater level was elevated within the entire contributing catchment area, however, this can be a misleading term, as the control system only occupied very little of the field (app. one m^2^ per regulation well). The hydraulic loading rate (HLR) to the systems differed substantially, as expected, being highest for DBR and lowest for CD due to the difference in size. Treatment of drainage water is a relatively new concept, as illustrated by that the oldest facilities were two 20-year-old FWS and the second oldest a 10-year-old DBR. The youngest and least studied measure was IBZ.

**Table 1:** Results from the process of finding and selecting relevant studies for free water surface constructed wetlands (FWS), denitrifying bioreactor (DBR), controlled drainage (CD) and saturated (SBZ) and integrated buffer zones (IBZ). WB: water balance

Drainage mitigation measure	Result of search	After screening title and abstract	Passing inclusion criteria	Study sites	Study years * replicates	WB score	Studies with low risk of bias of total sites (%)
FWS	7550	173	17	33	109	85	55
DBR	7550	173	9	19	54	83	21
CD	213	100	14	25	93	80	20
SBZ	187	24	1	6	19	83	17
IBZ	176	13	1	1	2	100	100

**Table 2:** Size of each type of drainage mitigation measure, catchment area, DMMCAR (ratio of facility area and catchment area), age and HLR (hydraulic loading rate to the facility area) for free water surface constructed wetlands (FWS), denitrifying bioreactors (DBR), controlled drainage (CD) and saturated (SBZ) and integrated buffer zones (IBZ). SD: standard deviation. For CD, age refers to study length

	Size		Catchment area		DMMCAR		Age		HLR mean ± SD (m)
	Mean ± SD (m^2^)	Range (m^2^)	Mean ± SD (ha)	Range (m^2^)	Mean ± SD (%)	Range (%)	Mean ± SD (year)	Range (year)	
FWS	5486 ± 9377	20–51 000	65.1 ± 220.2	0.8–971.0	1.8 ± 2.1	0.03–7.06	5 ± 5	1–20	20 ± 22
DBR	71 ± 46	15–128	10.9 ± 6.7	0.8–20.2	0.1 ± 0.1	0.04–0.38	4 ± 2	2–10	685 ± 647
CD	10 572 ± 29 590	1005–149 000	2.1 ± 4.3	0.1–14.9	100		4 ± 1	1–5	0.2 ± 0.1
SBZ	4229 ± 2802	460–7392	14.1 ± 14.5	3.4–40.5	7 ± 7.2	0.65–15.73	4 ± 1	2–6	6 ± 6
IBZ	250	250	15.0		0.2		1	1	99

### Free water surface constructed wetlands (FWS)

**Fig. 4:**
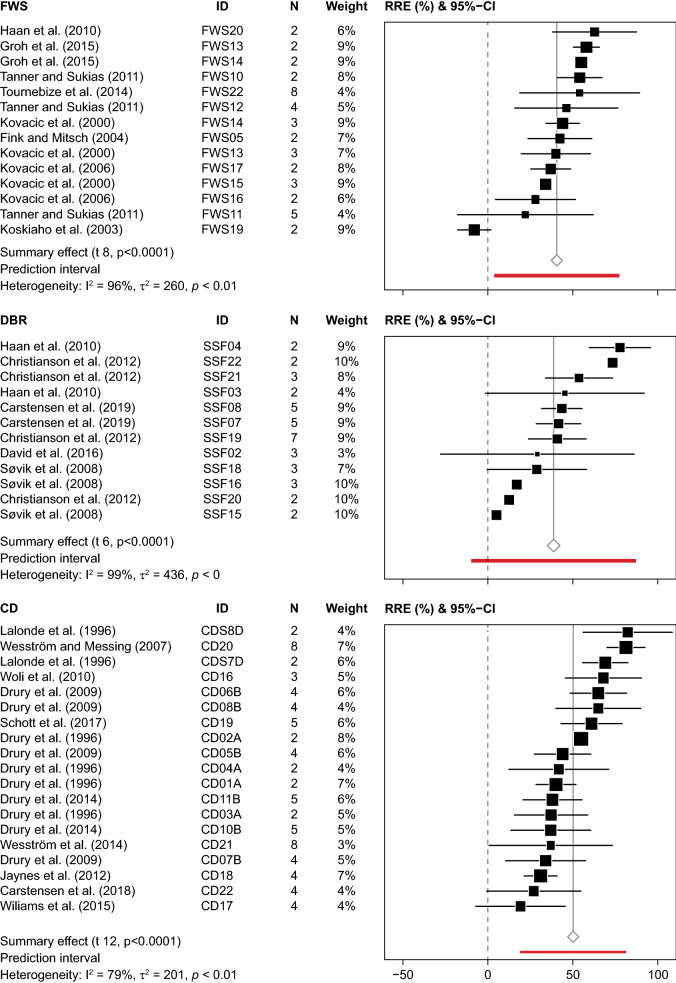
Forest plots showing effect sizes (RRE) and 95% confidence intervals (CI) of relative nitrate–N removal and summary effect with CI and prediction interval and heterogeneity analysis for free water surface constructed wetlands (FWS), denitrifying bioreactors (DBR) and controlled drainage (CD). N within-study sample size. ID represents FWS and DBR study sites; for CD the letter is unique for the research facilities

**Fig. 5:**
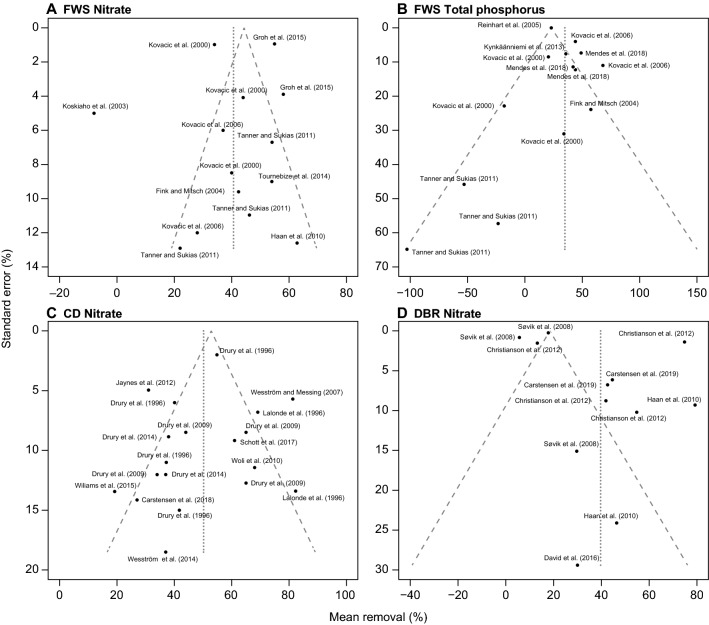
Funnel plots of free water surface constructed wetlands (FWS), denitrifying bioreactors (DBR), controlled drainage (CD) and saturated (SBZ) and integrated buffer zones (IBZ) for data sets containing results on nitrate–N or total phosphorus

The weighted average obtained by meta-analysis showed that FWS significantly reduced nitrate loading by 41% within a range from − 8 to 63% (Fig. 4). The CI varied from 29 to 51%, while the PI was rather broad, varying from 5 to 76%. The funnel plot did not indicate major biases, as the studies were more or less evenly scattered (Fig. 5). However, the heterogeneity of the selected sites was rather high (*I*^2^ = 96%), and *T*^2^ (260%) was higher than σ^2^ (70%). The subset analysis of data with either low risk of bias or sampling periods longer than two years/drainage seasons showed the average removal ranged between 40 and 44%, and CI and PI were slightly more narrow than for the full dataset (Table 4). Studies with N > 2 had lower *T*^2^, whereas σ^*2*^ was slightly higher, which lowered the heterogeneity. According to the arithmetic mean, the removal efficiency was 41% (CI: 29 to 51%) (Table 3). The absolute nitrate removal per FWS area amounted to 60 g N m^−2^ year^−1^ (CI: 29 to 91 g N m^−2^ year^−1^).

**Table 3:** Relative and absolute removal of nitrate–N, total phosphorus (TP) and total suspended solids (TSS) based on raw data and the meta-analysis for free water surface constructed wetlands (FWS), denitrifying bioreactors (DBR), controlled drainage (CD) and saturated (SBZ) and integrated buffer zones (IBZ). K^am^ is the number of study sites included when calculating the arithmetic mean, and K^meta^ is the number of study sites included in the meta-analysis

Drainage mitigation measure	K^am%^	K^meta^	Removal Mean ± SD (%)	Removal Mean^meta^ ± SD (%)	K^am^	Removal Mean ± SD (g m^−2^ year^−1^)
Nitrate–N						
FWS	18	14	40 ± 17	41 ± 21	21	60 ± 69
DBR	19	12	44 ± 21	40 ± 27	2	594 ± 481
CD	20	19	48 ± 18	50 ± 20	6	1 ± 1
SBZ	6		68 ± 39		13	23 ± 18
SBZ^a^	6		37 ± 25			
IBZ	2		26 ± 4		19	140 ± 50
TP						
FWS	16	15	18 ± 46	33 ± 28	8	0.68 ± 4.19
CD	7	7	29 ± 26	34 ± 32	2	0.03 ± 0.03
DBR	3		-50 ± 136		3	− 5.79 ± 20.96
IBZ	2		48 ± 6		20	2.44 ± 0.76
SS						
FWS	6		41 ± 16		6	1555 ± 936

^a^Includes the water and nitrate–N bypassing the SBZ

**Fig. 6:**
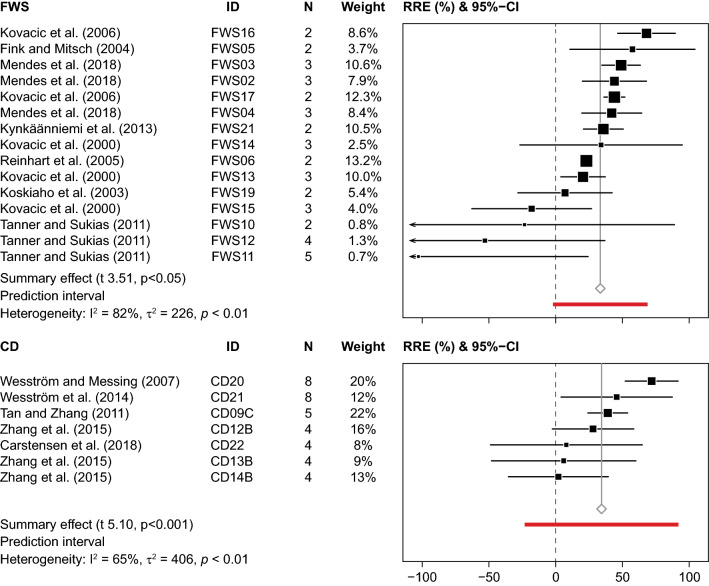
Forest plots showing effect sizes (RRE) and 95% confidence intervals (CI) of relative total phosphorus (TP) removal and summary effect with CI and prediction interval and heterogeneity analysis for free water surface constructed wetlands (FWS) and controlled drainage (CD). N within-study sample size. ID represents unique sites for FWS and DBR; for CD the letter is unique for the research facilities

According to the meta-analysis the average TP removal efficiency of FWS was 33%, ranging from − 103 to 68% (CI: 19 to 47%, PI: − 2 to 69%) (Fig. 6). The removal efficiencies did not follow a normal distribution; the data were skewed to the left due to net release of TP from multiple sites. The funnel plot showed an asymmetrical scatter of sites, as sites with TP release had much higher SE (Fig. 5). As expected, the heterogeneity was rather high, and T^*2*^ (226%) was much lower than σ^2^ (838%). The subset data analysis for TP removal reported a slightly higher removal (35%) than the initial data; however, σ^2^ was still very high as the included studies reported both removal and release of TP (Table 4). The data were further investigated by separating sinks and sources, showing that four sites exhibited a net release of TP (− 49%, CI: − 18 to − 83%) and eleven sites acted as sinks (38%, CI: 27 to 49%). The arithmetic mean TP removal efficiency was 18% (CI: − 4 to 46%) with an average absolute removal of 0.68 g P m^−2^ year^−1^ (− 1.16 to 2.52 g P m^−2^ year^−1^) (Table 3). The removal efficiency of TSS was 41% (CI: 28 to 54%) when calculated as the arithmetic mean.

**Table 4:** Results from meta-analysis of all data and data from sites with more than two years or drainage seasons (N > 2) and data from sites with low risk of bias (ROB) for free water surface constructed wetland (FWS), denitrifying bioreactors (DBR) and controlled drainage (CD). *k:* within-study sites, *SE:* standard error, *CI:* confidence interval, *PI:* prediction interval, *T*^*2*^: between-study variance, *σ*^*2*^: within-study variance, *I*^*2*^: proportion of unexplained variance

		Data analysed	*k*	SE	*t*	*p* <	Range	CI	PI	T^2^	σ^*2*^	*I*^2^	*Q* test (*p* <)
FWS	Nitrate–N	All	14	41	8	0.0001	− 8 to 63	29–51	5 to 76	260	70	96	0.0001
FWS	Nitrate–N	*N* > 2	6	40	11	0.0001	22 to 54	31–49	19 to 60	41	79	61	0.02
FWS	Nitrate–N	Low ROB	12	44	14	0.0001	22 to 58	37–51	14 to 74	175	66	96	0.0001
FWS	TP	All	15	33	5	0.0002	− 103 to 68	19–47	− 2 to 69	226	838	82	0.0001
FWS	TP	*N* > 2	8	35	2	0.06	− 102 to 49	20–49	− 29 to 81	373	1024	67	0.037
FWS	TP	Low ROB	14	35	5	0.0002	− 102 to 68	20–50	− 1 to 71	231	874	83	0.0001
DBR	Nitrate–N	All	12	40	6	0.0001	6 to 79	24–55	− 9 to 89	436	169	99	0.001
DBR	Nitrate–N	*N* > 2	6	35	7	0.0007	18 to 45	23–47	12 to 82	267	209	88	0.0001
DBR	Nitrate–N	Low ROB	4	35	5	0.02	13 to 45	10–59	− 58 to 127	408	41	94	0.0001
CD	Nitrate–N	All	19	50	12	0.0001	19 to 82	41–59	19 to 81	201	119	79	0.0001
CD	Nitrate–N	*N* > 2	13	49	9	0.0001	19 to 81	36–59	3 to 92	383	128	82	0.01

### Denitrifying bioreactors (DBR)

The weighted average calculated by meta-analysis showed a significant reduction of the annual nitrate loading by DBR of 40% within a range from 6 to 79% (CI: 24 to 55%, PI: − 9 to 89%) (Fig. 4). The funnel plot revealed asymmetry of data, where studies with low efficiency tended to have lower SE (Fig. 5). The heterogeneity analysis showed that the *I*^2^ was high (99%), as some of the studies were very precise, but showed different removal efficiency. Average *T*^2^ (436%) was much higher than σ^2^ (169%). The subset analysis of data with either low risk of bias or sampling periods longer than two years/drainage seasons reported lower removal efficiency (35%), and CI and PI were slightly narrower for studies with N > 2 (Table 4). Similar to FWS, studies with N > 2 had lower *T*^2^ and higher σ^2^. The arithmetic mean efficiency was 44% (CI: 35 to 53%), while the absolute nitrate removal per DBR volume amounted, on average, to 715 g N m^−3^ year^−1^ (CI: 292 to 760 g N m^−3^ year^−1^), ranging from 66 to 2033 g N m^−3^ year^−1^ (Table 4). This corresponded to an area-based nitrate reduction of 594 g N m^−2^ year^−1^ (CI: 333 to 855 g N m^−2^ year^−1^).

Only two studies included TP balances for the full drainage season, preventing meta-analysis. These two studies were somewhat contradictory in that one found release of TP (− 208% or − 30 g P m^−2^ year^−1^) and the other net removal (28% or 6 g P m^−2^ year^−1^) (Table 4).

### Controlled drainage (CD)

**Fig. 7:**
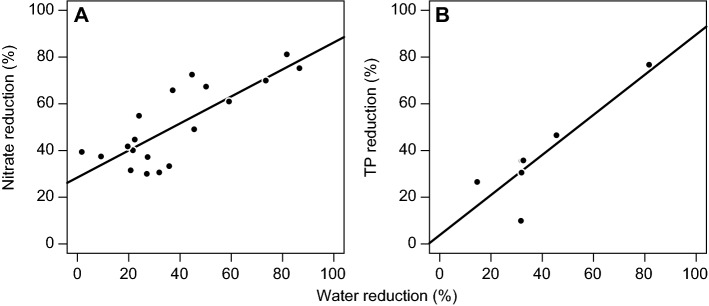
Percentage **A** nitrate and **B** total phosphorus removal vs. percentage reduction of drainage outflow at the outlet of fields with controlled drainage

The meta-analysis showed that CD significantly reduced the annual nitrate loading by, on average, 50% within a range from 19 to 82% (Fig. 4) (CI: 41 to 59%, PI: 19 to 81%). However, both CI and PI should be interpreted with care as the effect sizes did not follow a normal distribution. The funnel plot displayed a more or less even scatter of sites (Fig. 5). Heterogeneity was high (*I*^2^ = 79%), yet *T*^2^ was only slightly higher than σ^2^. The removal efficiency of studies including sampling periods longer than two years/drainage seasons was more or less similar to the result of the full data analysis (Table 4). However, the subset analysis pointed to the possible occurrence of two groups; one with a removal efficiency < 44% and one with a removal efficiency > 61%. The mean (arithmetic) nitrate removal efficiency was 48% (CI: 40 to 56%). The absolute nitrate removal amounted to 1.20 g N m^−2^ year^−1^ (1.16 to 1.24 g N m^−2^ year^−1^), corresponding to 12 kg N ha^−1^ year^−1^ (CI: 8 to 16 kg N ha^−1^ year^−1^). The relative nitrate reduction correlated well with the relative reduction of drainage flow (*R* = 0.80 (Pearson), *p* < 0.0001, *K* = 19), and exclusion of studies with sub-irrigation, a practice implying an additional water supply, improved this correlation (*R*  =  0.88, *p* < 0.0001, *K* = 10) (Fig. 7).

The average loss of TP via drainage water was reduced by 34% (CI: 10 to 58%, PI: − 23 to 92%) according to the meta-analysis (Fig. 6). The removal efficiencies did not follow a normal distribution as the data were slightly skewed towards the right. According to the statistical analysis, heterogeneity was moderate (I^2^ = 65%), while T^2^ (406%) and σ^2^ (403%) were more or less identical, suggesting that the studies were similar enough to justify combination. The arithmetic mean was 29% (CI 10 to 48%) (Table 4). The average absolute TP retention amounted to 0.03 g P m^−2^ year^−1^ (0.01 to 0.05 g P m^−2^ year^−1^) or 0.30 kg P ha^−1^ year^−1^ (0.10 to 0.49 kg P ha^−1^ year^−1^) (Table 4). The relative reduction of TP loading correlated well with the reduction of drainage flow (*R* = 0.87 (Pearson), *p* < 0.01, *K* = 6) (Fig. 7).

### Saturated and integrated buffer zones (SBZ and IBZ)

Removal efficiencies could not be aggregated using meta-analysis for the emergent technologies, SBZ and IBZ, as, until now, only one study containing multiple sites has been published for each practice (Table 4). The annual arithmetic mean removal efficiency was 75% (CI: 35 to 53%) of the nitrate loaded into the SBZ. However, between 6 and 77% of the water bypassed the SBZ; thus, taking all nitrate leaving the field into account, the average nitrate removal efficiency was 37% (CI: 17 to 57%) and varied from 8 to 84%. The absolute nitrate removal per SBZ area was 23 g N m^−2^ year^−1^ (CI: 9 to 37 g N m^−2^ year^−1^). There were no available data on TP balances for SBZ in the articles selected for this review. For IBZ, the annual nitrate removal efficiency, calculated as the arithmetic mean, was 26% (CI: 20 to 32%) (Table 4). The absolute nitrate removal per IBZ area was 140 g N m^−2^ year^−1^ (71 to 209 g N m^−2^ year^−1^). The removal efficiency of TP was 48% (CI: 40 to 56%), while the absolute TP removal per IBZ area was 2.4 g P m^−2^ year^−1^ (CI: 1.4 to 3.5 g P m^−2^ year^−1^).

## Discussion

### Removal efficiency and uncertainty of drainage mitigation measures

Removal efficiency was quantified in both absolute and relative values in our review. However, care should be taken when comparing values from different sites, as the absolute removal efficiency depended heavily on the nutrient loading to the system (Fig. S1). The loading rate of nutrients are highly site specific, as it is determined by the concentration of nutrients in the water and by HLR, which is highly variable from site to site. For example, for DBR, the specific loading rate of nitrate per DBR area differed substantially between sites (221 to 11,533 g N m^−2^ DBR year^−1^). Furthermore, the HLR varies from year to year, although this variation can be accounted for to some extent by monitoring over multiple years. In this review, it was demonstrated by that study sites monitored for multiple years (N > 2) had higher σ^*2*^, and thus incorporated more variation. Absolute removal was reported relative to mitigation measures surface area in our review, however, another possibility would be to report absolute removal per catchment area, however, the estimate of catchment areas are often very uncertain, adding more uncertainty to the removal estimate. The HLR also influence relative removal, where the removal efficiency tends to increase with decreasing HLR (Vymazal 2017; Hoffmann et al. 2019) (Fig. S2), though temperature is at least as important. The design of mitigation measures is commonly guided by DMMCAR, as a rough estimate of HLR and temperature; for instance, in New Zealand a guideline predicts that a DMMCAR of 5% will yield an approximate nitrate reduction of 50 ± 15% (Tanner et al. 2010), while in Denmark a ratio around 1–1.5% is recommended for FWS to ensure a HRT of minimum 24 h during winter (Landbrugsstyrelsen 2019). However, the optimal DMMCAR is site-specific and depends on hydrological and geochemical conditions, e.g. similar DMMCARs can have very different temperatures and HLRs (Fig. S3).

The quantification of nutrient loading and removal is somewhat uncertain as it relies on a black-box approach (i.e. input–output). This implies that the estimates depend especially on the frequency of nutrient sampling and the water flow monitoring strategy. Estimates of TP retention might be more uncertain than those of nitrate as TP concentrations in tile drainage water tend to change quickly over time, especially at high flow, which can be difficult to capture (Johannesson et al. 2017), whereas nitrate concentrations tend to change more gradually. Johannesson et al. (2017) tested the importance of flow monitoring strategy and found that TP retention was underestimated when based solely on outlet flow measurements rather than on both inlet and outlet flow measurements.

#### Free water surface flow constructed wetlands (FWS)

The results showed that FWS significantly reduced the nitrate loss from drainage systems. However, as expected, the efficiency varied considerably since the included studies differed in design (e.g. HLR, aspect ratio, size, carbon availability), age monitoring schemes and run off characteristics, factors that all affected the removal efficiency. At one site, nitrate release was reported, which was most likely due to the lack of monitoring of one of the inlets (Koskiaho et al. 2003), which emphasises the importance of the monitoring scheme. The removal efficiency found in this review was slightly higher than that of an earlier review, which reported a removal of 37% (CI: 29 to 44%) (Land et al. 2016). Compared with Land et al. (2016), the average absolute removal was much lower in our review (181 ± 251 g N m^−2^ year^−1^), which was not surprising, as their review included a broad range of created and restored wetlands treating both agricultural runoff, riverine water, secondary and tertiary domestic wastewater and urban stormwater. Our review of FWS showed that they did not always remove TP, as four out of 15 FWS sites acted as a source of P. This net release of P might be due to mobilisation of dissolved reactive P (DRP) from the sediment or the size of the FWS being too small to adequately decelerate the flow (Kovacic et al. 2000; Tanner and Sukias 2011). The studies reporting a net release of TP had a very high within-study variance and they were therefore given less weight in the meta-analysis, with the consequence that the removal efficiency was higher than the arithmetic mean. Both the relative and the absolute removal efficiency were lower compared with Land et al. (2016), probably because the average TP loading was much higher in the studies included in their review, where also FWS established in streams were represented. In our review, most studies on FWS had low risk of bias, although, often only the inlet or the outlet was monitored, which were compensated for in the studies by adjusting the unmonitored flow component with precipitation, evaporation or groundwater (if not lined with a non-permeable membrane).

#### Denitrifying bioreactors (DBR)

Our meta-analysis showed that DBR significantly reduced the nitrate loss from drainage systems to surface water. The removal efficiencies generally displayed high variations, which reflected the differences (e.g. design, age) between the studied sites, not least regarding nitrate loading rates. Many of the study sites were experimental facilities or pilot studies, implying that they were established to investigate and identify factors influencing performance. For example, among the studies included in our review, the low removal efficiency could be ascribed to short-circuiting within the system (Christianson et al. 2012a), inadequate sizing, i.e. too short HRT (David et al. 2016), and scarce monitoring (Søvik and Mørkved 2008). Accordingly, the average removal efficiency derived from the meta-analysis was most likely a conservative estimate since many of the sites with suboptimal design were given a relatively high weight due to low SE. Many of the DBR sites were assessed to have a moderate to high risk of bias, as flow was often only measured at either the inlet or the outlet, however, due to their small size, the uncertainty caused by this might be lower for DBR than for e.g. FWS.

#### Controlled drainage (CD)

According to our results, CD significantly reduced the loading of nitrate at the drain outlet. However, heterogeneity was relatively high and the efficiencies displayed high dispersion around the mean. This was expected, though, as the efficiency of CD is especially influenced by drain spacing and management, which differed between sites (Ross et al. 2016). For example, the target elevation of the water table differed considerably between sites, from 15 to 76 cm below the soil surface. The removal efficiency found in our review aligned very well with that from an earlier review of 48 ± 12% by Ross et al. (2016). The nitrate reduction was mainly regulated by the reduction of the flow at the drain outlet, which has also been stressed in earlier studies (Skaggs et al. 2012; Ross et al. 2016). Although many studies stated that CD was implemented to increase denitrification, higher denitrification rates or lower nitrate concentrations in drain water were seldom reported despite denitrification measurements (Woli et al. 2010; Carstensen et al. 2019a). This lack of denitrification was probably due to insufficient amounts of soil organic carbon, temperature limitation or absence of anoxic zones in the soil. Higher efficiencies could potentially be obtained if the water level was elevated even closer to the surface where the organic C content is higher, but this could increase the surface runoff (Rozemeijer et al. 2015) and/or harm the crop yield. The redirected water is either stored in the root zone or directed to alternative flow paths. If the excess water moves towards the stream without passing conditions suitable for denitrification, there will be no removal of nitrate and thus no effect of CD. In contrast, if the water passes deeper zones with reduced conditions or conditions favourable for denitrification, the nitrate will most likely be removed. Higher removal efficiency of CD could be gained if CD was combined with, for example DBR, treating the part of the water still leaving via the drainage system (Woli et al. 2010). A concern regarding the implementation of CD has been that the saturation of the root zone might cause desorption of redox-sensitive P, but none of the studies on CD reported TP or DRP release. However, in three studies the CI crossed the zero line, indicating that TP removal was not significant, which was supported by the PI. The retention efficiency determined in our study was considerably lower compared with Ross et al. (2016), who reported a TP retention of 55 ± 15%. Almost all sites with CD were categorised as having moderate to high risk of bias, as the majority of the studies only quantified the reduction in flow and nutrients at the drainage outlet. Only few attempted to quantify nitrate or P budgets for all flow paths leading nutrients to the surface water (Sunohara et al. 2014).

#### Saturated and integrated buffer zones (SBZ and IBZ)

Two novel technologies, SBZ and IBZ, were included in our review to demonstrate the recent development in this research area. Until now, SBZ have mainly been investigated in USA and with variable results (Jaynes and Isenhart 2019). Low performance of SBZ has been linked to selection of unideal sites containing permeable soil layers or sites where a low fraction of water was diverted to the SBZ, which is controlled by the length of the distribution pipe. Vegetation has also been argued to influence the efficiency of SBZ (Jaynes and Isenhart 2019) as higher removal efficiency has been found at sites with established perennial vegetation. This might be due to addition of more labile carbon to the soil to support denitrification or to enhanced immobilisation of microbial N by the more developed rhizospheres (Jaynes and Isenhart 2019). The removal efficiency of SBZ is difficult to quantify as the outlet of the SBZ is the riparian soil where N and P concentrations can only be measured with piezometers, and dilution by groundwater through flow can occur. Another concern is whether or not the piezometer measurements can be considered representative for the whole area. In our review, IBZ had the lowest average removal efficiency of the mitigation measures, which probably can be ascribed to that the two IBZs were experimental test facilities with too low DMMCAR and the vegetation was not fully developed (Zak et al. 2018). A recent technical report on IBZ showed that the removal efficiency of two full-scale facilities established in Denmark was 53–55%, which was even a conservative estimate (van’t Veen et al. 2019). The overall reduction of nitrate to the receiving water might be even higher than reported, as after passing the IBZ, the water infiltrates the riparian zone between the IBZ and the stream where nitrate can be further removed by denitrification or vegetation. Thus, more studies on SBZ and IBZ are needed to critically assess their nutrient removal efficiency and the uncertainty related to the monitoring of the outlet.

### Applicability in the farmed landscape

**Fig. 8:**
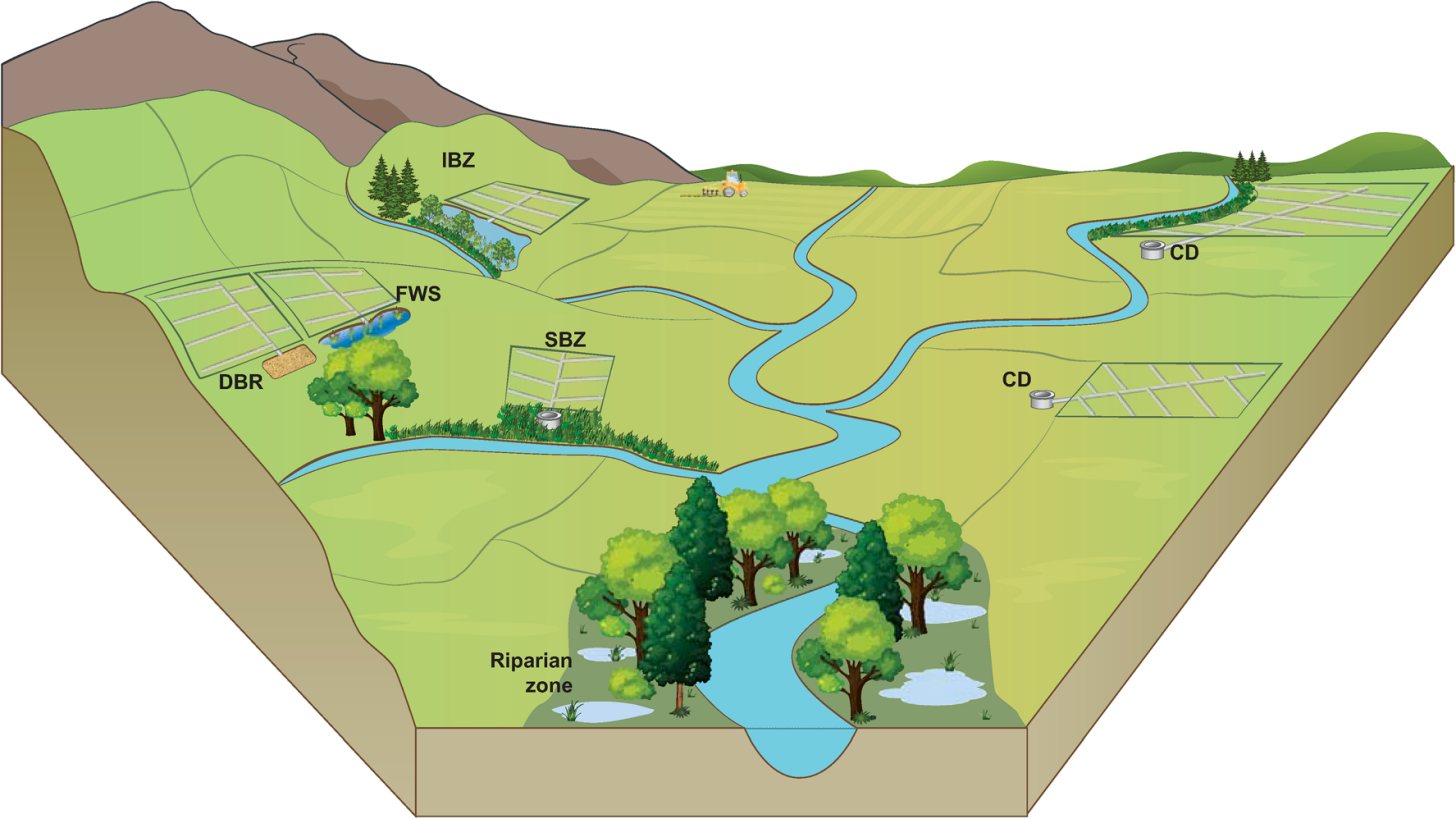
Conceptual diagram of potential locations of free water surface constructed wetlands (FWS), denitrifying bioreactors (DBR), controlled drainage (CD) and saturated (SBZ) and integrated buffer zones (IBZ) on mineral soils in a small catchment

The five drainage mitigation measures can seamlessly be integrated into landscapes with existing drainage systems, but to optimise performance and cost efficiency their individual applicability to the landscape must be evaluated carefully. Each measure varies in size and capacity to intercept water, where the size relative to the catchment area decreases in the order of FWS > SBZ > IBZ > DBR. Especially the size of the contributing catchment, slope and soil type determine how and where the measures can be implemented (Fig. 8). Flat landscapes (slope < 1%) are suitable for implementation of CD as a single control structure will affect a large area; however, as the technology advances it might soon be possible also to implement CD in sloping landscapes. In gently sloping terrains, FWS, DBR and SBZ fit as a hydraulic gradient is needed to move the water through the systems. The hydraulic gradient should preferably be minimum 2–3% for FWS and DBR, while for SBZ the slope of the landscape should be around 2–8% (Tomer et al. 2017). In addition, in sloping landscapes, IBZ are suitable as a hydraulic gradient of minimum 4% is required to move water through the pond and the infiltration zone (Fig. 8). In sloping areas, surface runoff is also more likely to occur, which can be intercepted by the IBZ (Zak et al. 2019).

Besides suitability to the landscape, implementation strategies are often guided by cost efficiency. Cost efficiency, including capital and operational cost of the drainage systems, has been calculated earlier (Christianson et al. 2012b; Jaynes and Isenhart 2019). However, the cost of preliminary examinations such as geological and soil investigations has often not been included despite that it can constitute a substantial part of the budget, and is therefore important to consider when selecting mitigation measure. Cost efficiency is inherently country specific since, as besides local costs, such as land acquisition, it depends on national regulation and implementation strategies. For example, in Denmark, FWS can only be implemented at a certain location if the catchment area is larger than 20 ha and if it removes more than 300 kg N ha wetland^−1^ year^−1^, and other requirements such as to soil clay content (> 12%) also prevail.

### Current advances in ecosystem service provisioning

The selection, implementation and design of drainage mitigation measures should ideally maximise the supply of ecosystem services and minimise undesirable by-products. Thus, the management and design of mitigation measures should not solely focus on nutrient reduction, but also take into consideration potential negative by-products, as some of these can be minimised by location or design (Carstensen et al. 2019b). For instance, DRP release and methane emission have been reported from facilities experiencing nitrate limitation (Robertson and Merkley 2009; Shih et al. 2011), while other processes need further investigation (e.g. nitrous oxide emission, loss of dissolved organic carbon). Permanent removal and recycling of P require plant harvesting or sediment removal; another course of action may be to combine mitigation measures with a P filter (Canga et al. 2016; Christianson et al. 2017).

The possibilities of optimising ecosystem services and synergies with the surrounding landscapes where drainage mitigation measures are applied are manifold (Goeller et al. 2016) e.g. biodiversity, water storage, phytoremediation (Williams 2002) or provision of biomass (Zak et al. 2019). Current examples of multiple ecosystem service provisioning are, the combination of CD, sub-irrigation and reservoirs, which according to Satchithanantham et al. (2014), can reduce the peak flow in spring and delay short-term water-related stress on crops in periods with less precipitation. In addition, sub-irrigation can increase crop yields (Wesström and Messing 2007; Jaynes 2012). According to our review, CD was combined with sub-irrigation at 14 out of 25 sites, while FWS were combined with a sedimentation pond at 6 of the 33 sites. A sedimentation pond is a simple supplement, which can increased the water storage capacity and give access to irrigation water and nutrients for recycling. Yet, the potential of mitigation measures for increasing the climate resilience of agricultural areas by retaining and storing more water in the landscape, thereby buffering hydrological peak events, needs to be investigated at catchment scale. Due to the potentials for adaptation and synergies with the surrounding landscape, these systems are innovative opportunities in future bio-economies, as the measures can reduce nutrient losses, while providing multiple ecosystem services e.g. nutrient reuse, biomass production, biodiversity, etc, if designed accordingly.

## Perspective: Opportunities and challenges for implementation of mitigation measures at catchment scale

Effective implementation of drainage mitigation measures requires a holistic approach encompassing both ecosystem services and potential negative by-products, while simultaneously maintaining a catchment scale perspective (Hewett et al. 2020). This require a catchment scale understanding of flow paths, taking into consideration all important transport paths influencing the quality of ground- and surface water (Goeller et al. 2016). Consequently, detailed information on local nutrient flow pathways and drainage systems is highly needed. It should also be emphasised that the mitigation measures discussed in this review only target drainage water, while other mitigation measures, such as cover crops, target the water before it leaves the root zone (Beckwith et al. 1998) or restored wetlands that target water further downstream (Audet et al. 2014). Consequently, it is essential that the drainage mitigation measures should complement and not compensate for farm management practices producing high pesticide, N or P leaching that influences other flow paths such as groundwater or surface runoff. Choosing the most appropriate and avoiding incompatible mitigation measures require collaboration between the different actors in the catchment to align the interests of all stakeholders (Hashemi and Kronvang 2020). To guide this decision process, we propose a further development of the sustainability index developed by Fenton et al. (2014), where weighting factors are assigned to relevant parameters. This index, serving as a tool for stakeholder involvement, could be expanded with more ecosystem services and cost effectiveness adapted to local conditions. Furthermore, application of a combination of mitigation measures may be more cost efficient than introducing only one option. In correspondence with this, a study by Hashemi and Kronvang (2020) found that it may be more cost effective to use a combination of targeted mitigation measures rather than a single option for reduction of the nitrate loading to aquatic ecosystems.

In addition to considering the local geographical and climatic conditions for selection and application of drainage mitigation measures, integration with future changes in climate and land use must be considered. Climate change is predicted to cause more intense and frequent precipitation events and prolonged summer droughts in the investigated climate regions (Christensen et al. 2013). The envisaged increase in temperature might improve the performance of the drainage mitigation measures, even though the intense precipitation events will challenge their hydraulic capacities and, thereby, their performance, potentially changing the need for mitigation measures at catchment scale. Human modifications of land use, land and water management induced by, for instance, a green shift to a new bio-economy (Marttila et al. 2020) might entail further expansion and intensification of land uses such as agriculture and forestry, which will increase the demand for drainage and thereby the need for implementation of drainage mitigation measures to reduce the nutrient losses.

## Electronic supplementary material

Below is the link to the electronic supplementary material.

Supplementary material 1 (PDF 232 kb)

Supplementary material 2 (XLSX 93 kb)
