# Supplementary material for: Efficiency of mitigation measures targeting nutrient losses from agricultural drainage systems: A review
Source: Ambio. 2020 Jun 3;49(11):1820–37. doi: 10.1007/s13280-020-01345-5 (PMC7502647; doi:10.1007/s13280-020-01345-5)
Supplement: Supplementary file 1 — Supplementary material 1 (PDF 232 kb) [file 13280_2020_1345_MOESM1_ESM.pdf]

Ambio

Electronic Supplementary Material

This supplementary material has not been peer reviewed.

Title**: Efficiency of mitigation measures targeting nutrient losses from agricultural drainage systems: a review**

Table S1. Search strings used for the search at Web Of Science 8 July 2019.

Mitigation measures	Search string
Free water surface constructed wetland and subsurface flow constructed wetland (FWS+SFF)	((bed OR bio*reactor OR bio*filter OR layer OR wetland) AND (nitrogen OR phosph* OR TP OR TN OR nitrate OR NO3* OR PO4* OR TKN OR sediment*) AND (retention OR trap* OR reduc* OR denitrification OR uptake OR sedimentation OR remov* OR settling OR accretion OR precipitat* OR *sorption OR loss OR performance*) AND (agricultur* OR drain*))
Controlled drainage (CD)	(“controlled drain*” OR “controlled tile drain*” OR “groundwater management” OR “drain* water management”) AND (nitrogen OR phosph* OR TP OR TN OR nitrate OR NO3* OR PO4* OR TKN) AND (remov* OR retention OR loss OR reduc* OR denitrification OR performance*) AND (agricultur* OR drain*)
Saturated buffer zone (SBZ)	(saturat* OR redirect* OR reconnect* OR flooding OR inundation) AND (“buffer strip*” OR “buffer zone*” OR “riparian strip*” OR “riparian buffer” OR “riparian zone*”) AND (nitrogen OR phosph* OR TP OR TN OR NO3* OR PO4* OR nitrate OR TKN OR sediment*) AND (retention OR trap* OR remov* OR reduc* OR loss OR denitrification OR uptake OR sedimentation OR settling OR accretion OR precipitat* OR *sorption* OR perfor*) AND (agricultur* OR drain*)
Integrated buffer zone (IBZ)	(integrat* OR ditch OR pond) AND (“buffer strip*” OR “buffer zone*” OR “riparian strip*” OR “riparian zone*”) AND (nitrogen OR phosph* OR TP OR TN OR NO3* OR PO4* OR nitrate OR TKN OR sediment*) AND (retention OR trap* OR remov* OR reduc* OR loss OR denitrification OR uptake OR sedimentation OR settling OR accretion OR precipitat* OR *sorption* OR performance*) AND (agricultur* OR drain*)

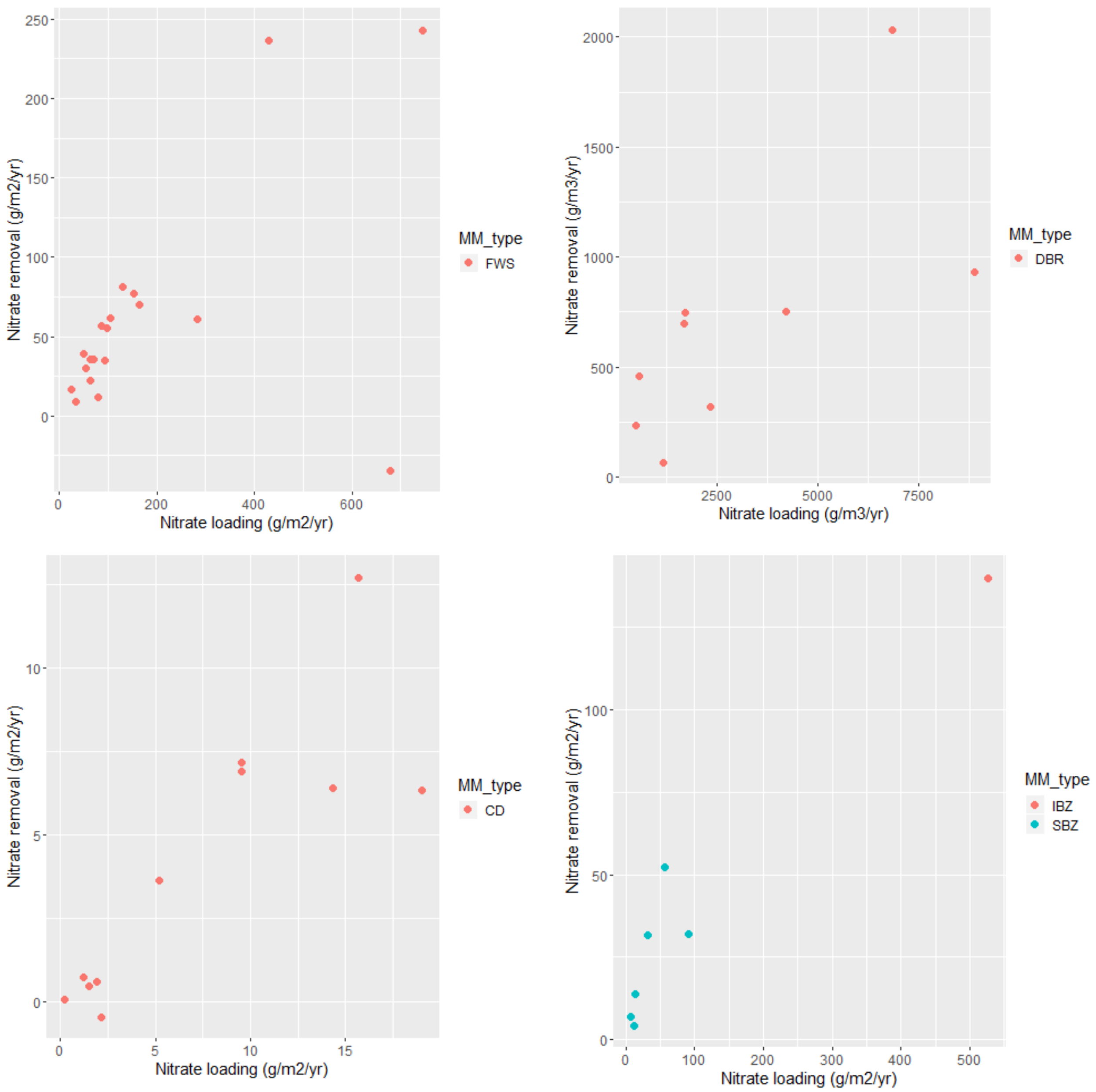

Figure S1. Nitrate removal rate (g m^{-2} yr^{-1}) vs. nitrate loading rate (g m^{-2} yr^{-1})

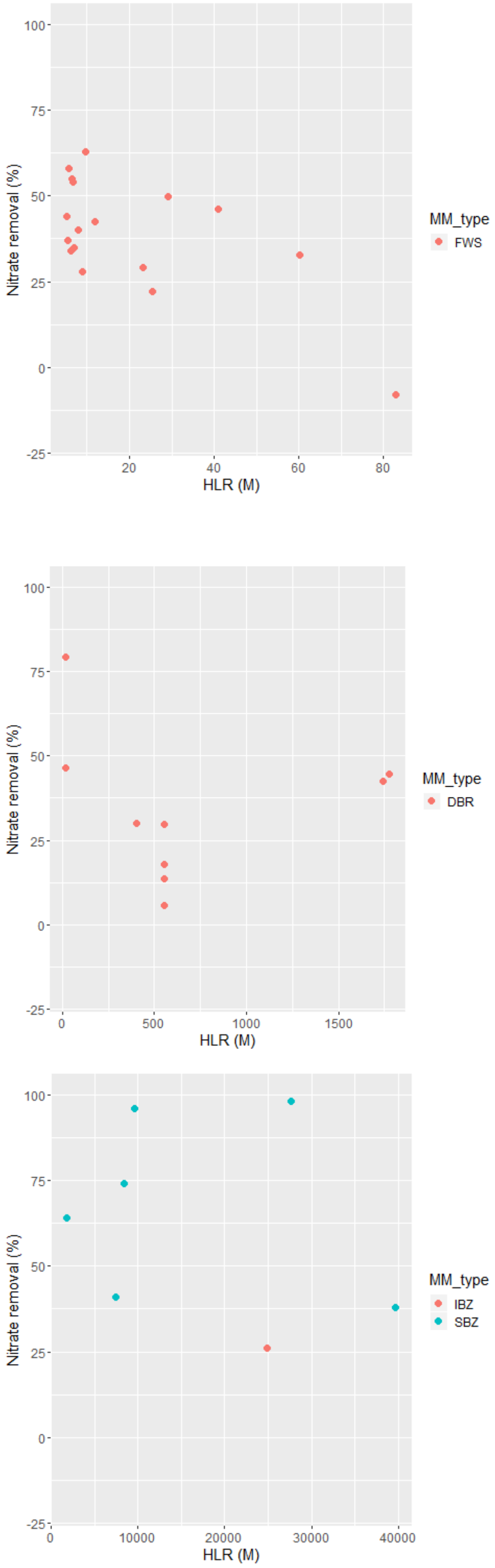

Figure S2 Nitrate removal efficiency (%) vs. hydraulic loading rate (HLR) (meter)

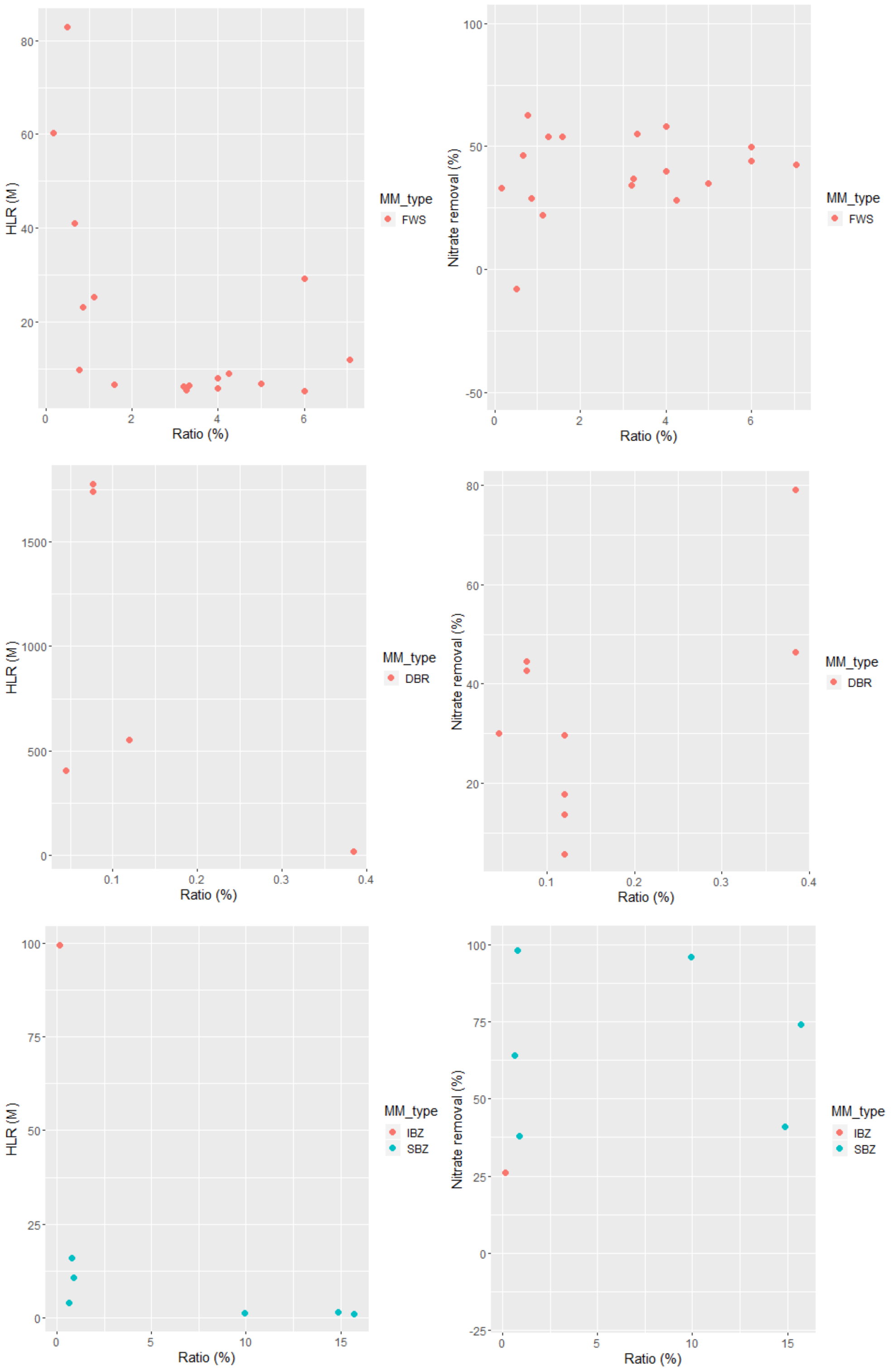

Figure S3. Nitrate removal efficiency (%) vs. facility to catchment ratio (Ratio) (%) and hydraulic loading rate (HLR) (M) vs. facility to catchment ratio (Ratio) (%).
